# JNK signaling dominance in hyperthermia

**DOI:** 10.1016/j.cstres.2025.100080

**Published:** 2025-05-06

**Authors:** Atsushi Enomoto, Takemichi Fukasawa

**Affiliations:** 1 Laboratory of Molecular Radiology, Center for Disease Biology and Integrative Medicine, Graduate School of Medicine, University of Tokyo, Tokyo, Japan; 2 Department of Dermatology, Graduate School of Medicine, University of Tokyo, Tokyo, Japan

**Keywords:** MAPK, ERK, JNK, Phosphorylation, Hyperthermia, Phosphatase, Cell death

## Abstract

Hyperthermia is a promising anticancer treatment that induces heat stress, stimulating various signal transduction pathways to maintain cellular homeostasis. Mitogen-activated protein kinases (MAPKs) link various extracellular stimuli with cytoplasmic and nuclear mediators through a three-tiered cascade of kinases, including MAPKs, MAP2Ks, and MAP3Ks. In mammals, three major groups of MAPKs have been characterized: extracellular signal-regulated protein kinases (ERK), p38 MAPKs, and c-Jun NH_2_-terminal kinases (JNK). Each group of MAPKs is heat-activated and exhibits distinct biological functions. However, the differences and advantages of the regulation of each MAPK with temperature changes remain unknown. Our results demonstrated that JNK was activated in a temperature-dependent manner, with degradation of the JNK phosphatases despite transient phosphorylation of ERK with induction of the ERK phosphatases. This brief insight deepens our current understanding of the deregulation of the ERK and JNK cascades in hyperthermia.

## Highlights

• Mild hyperthermia activates ERK1/2, but they are inactivated by induction of some ERK phosphatases above mild temperatures.
• JNK is activated in a temperature-dependent manner.
• JNK phosphatases, including DUSP4 and DUSP16, are degraded at cytotoxic temperatures, leading to sustained JNK activation.
• Above mild temperatures, expression levels of ERK and JNK phosphatases could be critical for their activation.

## Introduction

Hyperthermia is a treatment that raises the temperature of lesions using electromagnetic waves from outside the body and is often used in combination with radiotherapy or chemotherapy. In most hyperthermia treatments, the temperature is maintained above 42.5 °C to kill cancer cells. It increases cell temperature and induces various biochemical changes, such as reactive oxygen species (ROS) generation, an increase in intracellular calcium ion concentration, and protein degradation.^1^ Cellular proteins begin to denature above 42.5 °C. Hyperthermia-induced protein denaturation, aggregation, and degradation significantly disrupt cellular homeostasis.^2^

The mitogen-activated protein kinase (MAPK) signaling pathways play a crucial role in the response of cells to various extracellular stimuli, affecting cell proliferation, differentiation, survival, and death.^3^, ^4^ The MAPK signaling pathway consists of a three-kinase cascade, which includes MAPKs [extracellular signal-regulated kinase (ERK), p38, and c-Jun NH_2_-terminal kinases (JNK)], MAP2Ks [mitogen-activated ERK kinase (MEK) and MAP kinase kinase (MKK)], and MAP3Ks [e.g., mitogen-activated ERK kinase kinase (MEKK)]. MAP3Ks phosphorylate and activate MAP2Ks, thereby phosphorylating MAPKs. Biochemical and genetic analyses have indicated that MAP3Ks associate various extracellular stimuli with cytoplasmic and nuclear effectors by activating downstream MAPK signaling pathways.^5^ The ERK1/2 cascade is primarily activated by growth factors, cytokines, and G protein-coupled receptors that induce mitogenesis or differentiation.^3^ RAS is a type of small GTP-binding protein that interacts with the RAF family. At the cell surface, the activation of the RAS-RAF-MEK-ERK1/2 signaling pathway is initiated by ligand binding to receptor tyrosine kinases. RAF1 stimulates cell proliferation and facilitates cell survival by inhibiting apoptosis.^3^

The MAP3Ks of the JNK pathway include Ste20/Ste11/Ste7 family kinases such as apoptosis signal-regulating kinase 1 (ASK1), and members of the tyrosine kinase-like family, such as mixed lineage kinase and TGFβ-activated kinase-1 (TAK1). These MAP3Ks are predominantly activated by proinflammatory cytokines, endoplasmic reticulum stress, or extracellular stress.^4^ ASK1 facilitates ROS-induced and endoplasmic reticulum-mediated apoptosis.^6^ TAK1 plays a crucial role in regulating immune responses and cell survival through the NF-κB and JNK signaling pathways.^7^ Previously, JNK was confirmed as an apoptosis driver of cell death, implying its potential as a tumor suppressor.^3^, ^4^ Although studies have reported heat-induced activation of multiple MAPK signaling pathways,^8^, ^9^, ^10^ the differences in the regulation of each MAPK with temperature change remain unknown. In this report, we discuss how the advantage among multiple MAPKs is created in hyperthermia.

## Results

### Heat treatment stimulates differential phosphorylation of JNK and ERK

**Fig. 1:**
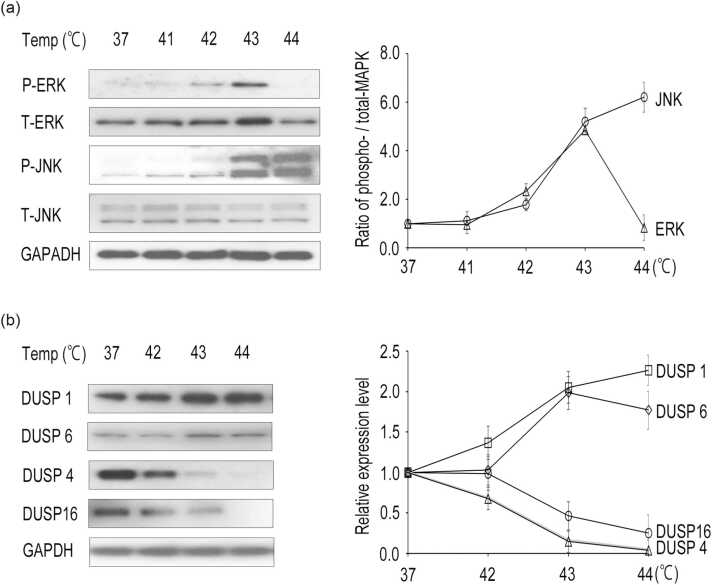
Dominance of JNK signaling above mild temperature. HeLa cells were heated at 41–44 °C for 1 h. Cell lysates were subjected to Western blot analysis. GAPDH was used as a loading control. The ratio of phosphorylated levels of ERK1/2 and JNK to the corresponding total protein (a) or relative expression levels of DUSPs to the control (b) was determined by measuring the optical density of band signals. Data are presented as means ± standard deviations of three independent experiments. Abbreviations used: DUSP, dual-specificity phosphatase; JNK, c-Jun NH_2_-terminal kinases. GAPDH, Glyceraldehyde 3-phosphate dehydrogenase.

MAPK pathways are activated by a wide variety of extracellular stimuli. Therefore, we examined the effects of heat on the expression and phosphorylation status of MAPK family members. Phosphorylation of JNK rapidly increased at 43 °C and above (Figure 1(a)). ERK1/2 phosphorylation gradually increased with heat treatment up to 43 °C but decreased at 44 °C. Heat treatment did not affect the expression of JNK but transiently increased the protein levels of ERK1/2. These results indicate that hyperthermia induces differential activation of JNK and ERK, suggesting the existence of different regulatory mechanisms in the JNK and ERK cascades.

### Differential expression of MAPK phosphatase after hyperthermia

We next investigated the effect of hyperthermia on the expression of MAPK phosphatases. The expression of dual-specificity phosphatase 1 (DUSP1), originally identified as an ERK phosphatase, increased in a temperature-dependent manner (Figure 1(b)). We also found that DUSP6, a specific phosphatase of ERK1/2, was induced with a peak at 43 °C. On the other hand, the expression of DUSP4, a JNK phosphatase, decreased in a temperature-dependent manner similar to that of DUSP16. These results indicate that heat-induced changes in DUSP1, DUSP4, DUSP6, and DUSP16 expression are consistent with changes in the phosphorylation status of ERK1/2 and JNK.

## Discussion

**Fig. 2:**
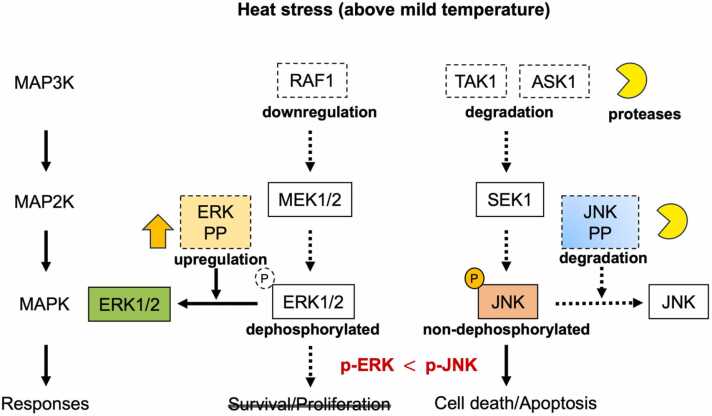
Schematic representation of the predominance of JNK signaling. Hyperthermia reduces MAP3K expressions in ERK and JNK pathways through degradation and downregulation, preventing MAP3Ks from activating downstream targets. Moreover, heat induces expression of ERK phosphatases, resulting in the dephosphorylation of ERK1/2. Conversely, JNK is activated by heat through degradation of JNK-specific phosphatases. This deregulated JNK activation could be one of the mechanisms underlying heat-induced cell death. Solid and dotted lines indicate activated and inactivated signaling, respectively. Abbreviations used: ERK, extracellular signal-regulated protein kinases; JNK, c-Jun NH_2_-terminal kinases; P, phosphorylation; PP, protein phosphatase.

Hyperthermia activates multiple MAPK signaling pathways.^8^, ^9^, ^10^ In various cell types, ERK1/2 activation facilitates cell survival. For example, mild heat treatment at 42 °C induces ERK1/2 activation, and the MEK-specific inhibitor PD98059 enhances heat-induced loss of viability by inhibiting ERK activation.^8^ Intense heat stress results in the dephosphorylation of ERK1/2 and Bcl-2, followed by Bcl-2 ubiquitination and thereby inducing apoptosis.^10^ Our results demonstrated that the phosphorylation of ERK1/2 increased at 42 °C and up to 43 °C but decreased at 44 °C (Figure 1(a)). These findings suggest that ERK1/2 is activated at mild temperatures (41–43 °C) but inactivated above 43 °C (cytotoxic temperature). Although the expression of ERK1/2 did not change above 43 °C, phosphorylation decreased sharply, indicating the potential involvement of ERK phosphatases. A subfamily of DUSPs contains MAP kinase-binding or kinase-interacting motifs that regulate the magnitude and duration of MAPK/JNK signaling pathway transduction by dephosphorylating their substrates.^11^ Acute heat shock at 43 °C increases the gene expression of DUSP1, a MAPK phosphatase, in human peripheral blood mononuclear cells.^12^ We also found that the expression of DUSP6 increased at higher temperatures, as did DUSP1 (Figure 1(b)), leading to the dephosphorylation of ERK1/2. Conversely, acute phosphorylation of both ERK1 and ERK2 is induced in DUSP1/6 inhibitor-treated CLL cells.^13^ Thus, our results and other reports suggest that the dephosphorylation of ERK1/2 at cytotoxic temperatures is mediated by the induction of DUSP1/6. Furthermore, our previous reports demonstrated that hyperthermia reduces the expression and kinase activity of several MAP3K members, such as RAF1, without reducing the downstream components in the ERK cascades.^14^ Additionally, heat shock failed to stimulate the activation of RAF members, including A-RAF, BRAF, and RAF1.^8^ Therefore, hyperthermia above mild temperatures inactivates RAF members, blocking the transmission of proliferative stimuli into activation signals and signal transduction through phosphorylation. This leads to the inhibition of cell survival and antiapoptotic signaling. Thus, under milder heat shock conditions, ERK1/2 activation proceeded mainly through the RAF-MEK-ERK signaling pathway, whereas under more severe heat shock, RAF1 activation was inhibited due to its downregulation, and the induction of ERK phosphatases could be crucial (Figure 2).

In contrast to ERK1/2, JNK is activated up to 45 °C in a temperature-dependent manner.^11^, ^15^ Our previous report demonstrated that DUSP16, a JNK phosphatase, was degraded in a proteasome-dependent manner by hyperthermia, leading to deregulated JNK activation.^16^ In this study, we showed that DUSP4, a negative regulator of JNK, decreased in a temperature-dependent manner (Figure 1(b)). While DUSP4 and DUSP16 can bind to all three MAPKs, they preferentially inactivate JNK.^11^, ^17^ Recent reports showed that DUSP4 or DUSP16 inhibits JNK activation, thereby reducing apoptosis and promoting cancer chemoresistance.^18^, ^19^ Another report indicated that the JNK phosphatase M3/6, also known as DUSP8, is inactivated by heat shock at 45 °C.^15^

Hyperthermia stimulates the release of cytokines, leading to the activation of TAK1. Additionally, treatment with heat increases ROS generation, resulting in the activation of a ROS-responsive protein kinase, ASK1. We recently demonstrated that hyperthermia at 41–43 °C reduced the expression and kinase activity of TAK1 and ASK1 in the JNK pathway through calpain-dependent or proteasome-dependent protein degradation.^14^, ^16^ The JNK signaling pathway is often associated with pro-apoptotic effects. Dominant-negative JNK prevents heat-induced cell death and translocation of Bcl-2-associated X protein (Bax) to mitochondria.^20^ Thus, under milder heat shock conditions, JNK activation proceeded mainly through the JNK cascade, whereas under more severe heat shock, MAP3Ks such as ASK1 and TAK1 could no longer be activated, making the inhibition of JNK phosphatases critical. There are alternatives, such as the involvement of certain heat-resistant MAP3Ks, excluding the aforementioned kinases.

A precise balance between the activation and inactivation of the MAPK signaling pathway must be strongly regulated to maintain cellular homeostasis. The efficiency of hyperthermia depends on the temperature and duration of heat treatment, which affects the activity of each MAPK. During mild hyperthermia, ERK1/2 may be active, aiding in cell survival and apoptosis inhibition. Hyperthermia at cytotoxic temperatures can disrupt the balance between ERK and JNK activation through decreased MAP3K expression, induction of ERK phosphatases, or reduced JNK phosphatase function, resulting in the amplification and predominance of JNK signaling (Figure 2). Therefore, cellular homeostasis in hyperthermia may be maintained through the regulation or deregulation of MAPK signaling at different temperatures.

## Conclusion

This study showed that severe temperatures induced the expression of ERK phosphatases while decreasing the expression of several JNK phosphatases. Such changes in expression of MAPK phosphatases could be one of the mechanisms by which JNK signaling becomes dominant.

Inhibition of ERK signaling and JNK phosphatases induces apoptosis. Thus, targeting MEK1/2 and DUSP4/16 may provide a more effective approach for cancer therapy.

## Materials and methods

### Cell culture and stimulation

Human cervix epithelial carcinoma HeLa cells were cultured as described previously.^14^ Hyperthermic treatment was conducted at 41–44 °C for 60 min by inserting a catheter equipped with an 8-MHz capacitive heating device (Thermotron RF-8; Yamamoto Vinita Co, Ltd, Osaka) into the culture flask with an average power of 4.3 to 5.1 W.

### Western blot analysis

Western blot analysis was performed, as described previously.^14^ The blots were then incubated with one of the following antibodies: anti-JNK (Cell Signaling Technology, Beverly, MA), antiphospho-JNK (Cell Signaling Technology), anti-ERK1/2 (Proteintech, Rosemont, IL), antiphospho-ERK1/2 (Proteintech), anti-DUSP1 (Merck, Darmstadt), anti-DUSP4 (Proteintech), anti-DUSP6 (Proteintech), anti-DUSP16 (Proteintech), and anti-Glyceraldehyde 3-phosphate dehydrogenase (MBL, Tokyo). The resulting signals were detected on X-ray films (GE Healthcare, Buckinghamshire) using an enhanced chemiluminescence detection system (GE Healthcare). The captured images were analyzed using ImageJ (NIH, Bethesda) and quantified by measuring the density of each protein band.

## Ethics statement

Not applicable.

## Funding and support

This work was supported, in part, by grants from the Ministry of Education, Culture, Sport, Science, and Technology of Japan (20K08100) and by the Japan Health and Research Institute.

## CRediT authorship contribution statement

**Takemichi Fukasawa:** Writing – review & editing, Validation, Formal analysis. **Atsushi Enomoto:** Writing – original draft, Visualization, Supervision, Project administration, Investigation, Funding acquisition, Formal analysis, Data curation, Conceptualization.

## Declarations of interest

The authors declare the following financial interests/personal relationships which may be considered as potential competing interests: TF belongs to the Social Cooperation Program, Department of Clinical Cannabinoid Research, supported by the Japan Cosmetic Association and Japan Federation of Medium & Small Enterprise Organizations. Other authors have declared that no conflicts of interest exist.

## Data Availability

Data will be made available on request.
